# Supplementary material for: Patterns of Intron Gain and Loss in Fungi
Source: PLoS Biol. 2004 Nov 30;2(12):e422. doi: 10.1371/journal.pbio.0020422 (PMC532390; doi:10.1371/journal.pbio.0020422)
Supplement: Table S1 — Also available at http://genes.mit.edu/NielsenEtAl/. (4.3 MB ZIP). [file pbio.0020422.st001.zip › NielsenEtAl/html/1302.html]

AN1980.1.NCU08499.1.MG03253.1.FG00632.1


```
 CLUSTAL W (1.82) Multiple Sequence Alignments - Introns Inserted


Sequence 1: NCU08499.1	951 aa
Sequence 2: MG03253.1	942 aa
Sequence 3: FG00632.1	995 aa
Sequence 4: AN1980.1	883 aa
Alignment Length: 1070 aa
Number Identitical Residues: 286 aa
Alignment Score (without introns) 15136


MG03253.1 	------MADQVNT----------------TTTESTAPVTSEPVRPVS-------------
NCU08499.1	-----MSASEVQEGAAAP-----------HEAVATKEMPTETVQEVT-------------
FG00632.1 	MSQPQEVASPVIESHTSPDPVPELEKQEKQETHDTEEKHDAPVMEEKKDIPEIPDKQDIT
AN1980.1  	-----MSAG---------------------------------------------------
          	       *.                                                   

MG03253.1 	----------------------PASD-SDKET0DNFEDAPDTASIRSLTRRKPSTTQPIS
NCU08499.1	----------------------HEHEKEDAAS~SPVTETKDKDSQPDHQRPASLAPTNLS
FG00632.1 	LDMEDKPGDEEINEKTDERIEKPETKIEEKLE~DKFEDSSEDDSDSDIGKKQEQKVDEKP
AN1980.1  	------------------------TENRDYEG0DSFHDSSE----------APNAVNNLS
          	                         .  :    . . :: :                  .

MG03253.1 	EDNQSEL~AAAFNKIKN---KKVAKDQGPS~NATKDKETEKEVTNADEATEPAAVSLSPT
NCU08499.1	DPDDKDT0DTFEDAIDTGSVRSLTKRSSSF~HRPRSPSMDDSAAADADHDARSHTTETPQ
FG00632.1 	EEKPEEK~PSQNDVNIKRLSSGMESDDSVS0ASDTDDKFEDAVAQSPTRSLTKRTISHVK
AN1980.1  	LSIPQSA~ST----------RSLTDSPPSA~SNATSPQFTEATQLPRTPDDDSKTDDEFH
          	    ..   :            : .          . .  . .           .     

MG03253.1 	VP-PVSEPAVDNVEKLSEPLGISEPNSKRISKASTNGELNEVNLNDKEETNPPVEEPLAQ
NCU08499.1	VP-KTAEIKEEEQEDVFETP-AQEQESKSLHRISHASKVSEASAFSAASLD---DVSLND
FG00632.1 	APSSSTEEQVDRRSEESYTREDTQEPSSAHHKATASESGSIASSIRERQARRKPSVASSR
AN1980.1  	ETGADGQSEAEATPRKQKLQKSPLLTAHRLSTSSLDEVNLTGNLNNDGSLD---HFPSNQ
          	 ..   :   :               :      :        .     .       .   

MG03253.1 	TPTIKNTPP~PTVAADEKP---~---VPARESPKTLSLSSITNALPAMPWSPPATESPTK
NCU08499.1	NSTIGDKTE1KSLDT-------~-----APSSTKKLSISSIASALPAMPWSPSATDEAVK
FG00632.1 	ISNTANLDN~VSLDDDTPPSPL1PKDLPPKPSSKTISLNSIS--LPSMPWSPAAEPAKSP
AN1980.1  	ETELGSPPA~LPSRD-------~------STSTQSSLLQGLSGSLPSVPWAP--------
          	 .   .     .                   *.:.  :..::.:**::**:*        

MG03253.1 	-------AMAINPQPSIPPPITAPPPPAPATRKLTSPFSWLSRNSTKDKEAPAPPPVS--
NCU08499.1	SPPAAAAAAAAAAPPAPAPAALTPAPPPPVTRKLTSPFSWLSRSSSKEQANPPPPPAAAT
FG00632.1 	----------VPPTATLAPSPPAPQPPPPPSRKLTSPFSWLSRSSSKEREPPLPPPPT--
AN1980.1  	------------PPQNKHPPPAAPPPPP--TRKITSPFAWLSRASTGSKDAVQS------
          	            .     *.  :* **.  :**:****:**** *: .:    .      

MG03253.1 	-PRRNTASSVTTLTSNPD-ILGRLEEEGKNGTAA-----RNTLKDRFKLVRMREEAGITL
NCU08499.1	APRRNTASSVATLSSNPDGTLGKVDEEGN----------KNTLKDRFKQLRLREEGSAPT
FG00632.1 	-GRRNTAGSIATLASNPEMMLSRLEEEKDGDSLPGDPVQRVTLQDRFKQLRQQEEAARAL
AN1980.1  	-GRRNTAASISTIGSNPDIAGRSQDLEVDGSSVGSRKPQRNSLKDQFKLLRMREEGHGPE
          	  *****.*::*: ***:      : * ...:  .   .: :*:*:** :* :**.  . 

MG03253.1 	SEDDKSPV---ATKSSTGVTSPVSNTSTDDKDS----LAVQQPPTSPLPGSPNKSLAPGT
NCU08499.1	GDDDE--------KSST----------PEEKE------AVQ-PPPSPLPPTPNPALAPGT
FG00632.1 	TGGENS-------KYDG----ALGLTLPDDGQ------TQDKPPLSPVP-QPISGLAPGT
AN1980.1  	TDEASVASGRTSGRASISHSVASPPSIPEEGENDVTAALPAVSPSTPPTSTVNPNLPPGT
          	    . . . :: : . . : .   : .:: :.. ::     .* :* .      *.***

MG03253.1 	ASGVDAAIVD--GQPVDWDLWQSLVYEGHAAVARTSSEELNRAIAVGIPNVIRGLVWQTL
NCU08499.1	ASGVTAGPAGS-DTPVDWDLWQTVVYEGPAAVARTSPEQLKEAIAKGIPSAIRGVIWQVL
FG00632.1 	VSGVNAGPSAISEEKVDWDLWQSVVYEGPTAVARTSAEELNKAIATGIPSAIRGVVWQVL
AN1980.1  	VSGVSASASDA-AAPVDWELWQQLVNEGPQVLKGTNSEELNAAIKRGIPQTIRGVIWQIL
          	.*** *.        ***:*** :* **  .:  *..*:*: **  ***..***::** *

MG03253.1 	AQSKNEGLEHVYRELVARGTDKEKPNNRQSGSSVGTASNTSNLSIQQRGETASSASSVNS
NCU08499.1	AQSKNEELETVYRDLVARGTDKERKTDRQSNASIS----LSNGGKPSGKDTPSSASSVHS
FG00632.1 	ADSKNDDLELVYKELATRGADKSK--DRNSNSTTASSLSNGNLSVHSGDAVASSASSINS
AN1980.1  	ADSRNLELEEVYKELIARGTDREK--SRANGMANGCAERDSAASSRSSTRSERSISAAPS
          	*:*:*  ** **::* :**:*:.:  .* .. : . :   .  .  .      * *:  *

MG03253.1 	D-GGSSGGAAPAVDSKEAEAAA---ARLLAERKKREKEEKAALA---KLEKVIRKDLGMR
NCU08499.1	EKSGPNGTGAPSPTEKDAEALAKAQALAAAERKKKEKEDKEMLS---RLEKVIRRDLGAR
FG00632.1 	DNSGNGTTSPP--NEKNAEAVLKAQVAAAAERKRKDKEDAASLQ---KLEKTIRRDLGAR
AN1980.1  	N----SNSSPSTPHEQDPEKLAKEQVAQETARKKKAKEDAAALQKLQKLEKAIRRDLGAR
          	:    .  ...:  .::.*   . ..   : **:: **:   * . .:***.**:*** *

MG03253.1 	TSFSKFAASQGLQEGLYGVCKAYALFDEAVGYAQGMNFLVMPLLFN0MPEEEAFCLLVRL
NCU08499.1	TSYSKFAAAQGLQEGLFGVCKAYALFDEAVGYAQGMNFLVMPLLFN~MPEEEAFCLLVRL
FG00632.1 	TSYSKYAAAAGLQEGLFGVCKAYALFDEGVGYAQGMNFLIMPILFN0MPEQEAFCLLVRL
AN1980.1  	TSYSRYFVSQGNQEGLFGLCKAYALYDEAVGYAQGMNFIVMPLLFN0MDEAEAFTLLVKL
          	**:*:: .: * ****:*:******:**.*********::**:*** * * *** ***:*

MG03253.1 	MNHYHLRDLFIHDMPGLHKNLYLFERLIEDLEPALYCHLHRKGISSHLYATQWFLTLFAY
NCU08499.1	MNQYHLRELFIQDMPGLHKCLYQFERILEDLEPALYCHLHRRGISPHLYATQWFLTLFAY
FG00632.1 	MNHYKLRDLFIQDMPGLHMHLYQFERLLEDFEPALYCHLHRKGISPHLYATQWFLTLFAY
AN1980.1  	MNQYGLREMFIQDMPGLHRCLYIFERLLEDFEPALYCHLRRRGVHPQLYATQWFLTLFAY
          	**:* **::**:******  ** ***::**:********:*:*: .:*************

MG03253.1 	RFPLQLVLRIYDLILSEGLS-AILKFGVVLMQKNAAALLQISDMSALTAFLRDRLFDVYI
NCU08499.1	RFPLQLVLRIYDLIFSEGLS-AIIKFGIVLMQKNATALLGMSDMSQLTTFLKDRLFDVYI
FG00632.1 	RFPLQLVLRIYDLIFSEGLS-AILRFGIVLMQKNASILLGLSDMQQLTTYLKDKLFDVYI
AN1980.1  	RFPLQLVLRIYDLILEEGLESTILKFALAIMRRNSDALLAMKDMAPLTAFLKDRLFDVYI
          	**************:.***.::*::*.:.:*::*:  ** :.**  **::*:*:******

MG03253.1 	DVAPSAGGILENGFFGSSSSNIDKEVYRADQMVRDACEVKITPEILAAYTEEWEEKTAAE
NCU08499.1	DATPSSNSILENGFFGSSSASIDKEVYRADQLVRDACDVNITAELLKEYGKEWEEKTKAE
FG00632.1 	DKDPSQGSLLENGFFGSSSSSMDKEVYRADQLVKDACEVQITPEMLKAYSTEWEEKTKAE
AN1980.1  	DKQPTPSSILESGFFGSS-GAADKEIYRADIMVQDACDIPLDPATINAYTAEWEEKERSE
          	*  *: ..:**.****** .  ***:**** :*:***:: : .  :  *  *****  :*

MG03253.1 	RQREAEITRRDAEIEELRAINTASAFKIRRLEEQLQVHDSEQAALATQLVHFKVENQELR
NCU08499.1	KER-------EQELEGLKQANTNYTVIVRRLEERIEAVDREQASLATELVRTKVENEELR
FG00632.1 	KDR-------ETELLELRSANHSFSIRLRKMEERVEACDREQADLATELVHTKVENQELK
AN1980.1  	KER-------EAELNHLRHTVATQSARIRLLEEQAEASDKEHVQLASEVVHLKVENEELS
          	::*       : *:  *:      :  :* :**: :. * *:. **:::*: ****:** 

MG03253.1 	DEAESLREQTKTLRTLLEEQPTELEKAWTEEKQGLLAKMDKVHEENRRIQQEMSDMEQQL
NCU08499.1	DENESLRGQVKELRVVIEHQSTEMENKWNLERDDLMKRNEKVHQENQKLEKELADLEEQL
FG00632.1 	DENDSMRGQVRELKIVIEKQPMDLEERWKLERDDLMKRNMHVHEENQRLEKELGELEEEL
AN1980.1  	DINDALRMQVKELKIVVDQQPAEVEQKLQTEMDRIMKRNVEVQNENRAMVEQMAEMEKEL
          	*  :::* *.: *: :::.*. ::*:    * : :: :  .*::**: : :::.::*::*

MG03253.1 	VKTKMEYAE0IN-------------------------SQHETLTRRWTDLKRQFA--
NCU08499.1	VQTKLQYAE~VSSYISCQGQNNRDNEKNANSFYEQINSAHETLTRKWADLKRQVQAA
FG00632.1 	VQTKLRYAE0IN-------------------------SQHETLNRKWTDLKRQFA--
AN1980.1  	VAAKLSYAE0IH-------------------------EQYEAQKQKWNDIRKALD--
          	* :*: *** :                          . :*: .::* *::: .
```
